# Full-Length Transcriptome Sequencing Reveals Tissue-Specific Gene Expression Profile of Mangrove Clam *Geloina erosa*

**DOI:** 10.3389/fphys.2022.851957

**Published:** 2022-04-20

**Authors:** Xin Liao, Yunqing Liu, Tingyu Han, Mingliu Yang, Wenai Liu, Yadi Wang, Chunpeng He, Zuhong Lu

**Affiliations:** ^1^ Guangxi Key Lab of Mangrove Conservation and Utilization, Guangxi Mangrove Research Center, Beihai, China; ^2^ State Key Laboratory of Bioelectronics, School of Biological Science and Medical Engineering, Southeast University, Nanjing, China; ^3^ Henan Key Laboratory of Big Data Analysis and Processing, Institute of Data and Knowledge Engineering, School of Computer and Information Engineering, Henan University, Kaifeng, China

**Keywords:** mangrove, *Geloina erosa*, SMRT, full-length transcriptomics, gill, hepatopancreas, muscle

## Abstract

Mollusca is the second largest animal phylum and represents one of the most evolutionarily successful animal groups. *Geloina erosa*, a species of Corbiculidae, plays an important role in mangrove ecology. It is highly adaptable and can withstand environmental pollution and microbial infections. However, there is no reference genome or full-length transcriptome available for *G. erosa*. This impedes the study of the biological functions of its different tissues because transcriptome research requires reference genome or full-length transcriptome as a reference to improve accuracy. In this study, we applied a combination of Illumina and PacBio single-molecule real-time sequencing technologies to sequence the full-length transcriptomes of *G. erosa* tissues. Transcriptomes of nine samples obtained from three tissues (hepatopancreas, gill, and muscle) were sequenced using Illumina. Furthermore, we obtained 87,310 full-length reads non-chimeric sequences. After removing redundancy, 22,749 transcripts were obtained. The average Q score of 30 was 94.48%. In total, 271 alternative splicing events were predicted. There were 14,496 complete regions and 3,870 lncRNAs. Differential expression analysis revealed tissue-specific physiological functions. The gills mainly express functions related to filtration, metabolism, identifying pathogens and activating immunity, and neural activity. The hepatopancreas is the main tissue related to metabolism, it also involved in the immune response. The muscle mainly express functions related to muscle movement and control, it contains more energy metabolites that gill and hepatopancreas. Our research provides an important reference for studying the gene expression of *G. erosa* under various environmental stresses. Moreover, we present a reliable sequence that will provide an excellent foundation for further research on *G. erosa.*

## Introduction

The mangrove clam *Geloina erosa* syn. *Polymesoda erosa*, syn. *Geloina expansa* (Twaddle et al., 2017; Yahya et al., 2018), belongs to Mollusca, Bivalvia, Heteodonta, Veneroida, Corbiculidae, Gelonia (Ingole et al., 2002), and is a type of large clam (Morton, 1976; Yingya et al., 1995). It is widely distributed throughout the Indian ocean and pacific coast and mainly inhabits mangroves, as well as estuaries and brackish marshes (Morton, 1976; Yingya et al., 1995). *G. erosa* plays an important role in the macrobenthic community in the mangrove habitat of the estuary. It is also the main target of mangrove tidal flat fisher. Despite this, there is little research on this species. *G. erosa* can use its mantle margin to achieve aerial respiration and its mantle cavity to absorb subterranean water during long-term exposure to the shore (Morton, 1976). They also take up pollutants present in the surrounding environment, which makes them excellent bioindicators of heavy metals and organic pollutants in mangrove environments (Modassir, 2000; Lai et al., 2011; Xing et al., 2017; Bertucci et al., 2018; Ma et al., 2019; Almeida et al., 2020; Wang et al., 2020; Zhang et al., 2020). In addition, *G. erosa* has important economic and medicinal value, Studies have found that the extract of *G. erosa* has high antiviral activity against influenza virus strains type-A (A/Missisipi 1/85/H3N2) and type-B (B/Harbin 7/94) (Morton, 1985; Chatterji et al., 2002).

To fully understand the biology of *G. erosa*, it is necessary to study the underlying genes that contribute to its functions and physical characteristics. Liu et al. (2021) published the world’s first comprehensive mollusc genome database, which provides a valuable resource for studying the genome of mollusks. Zhao et al. (2017) studied tissues, from the gills and the hepatopancreas during external infections in *Sinonovacula constricta*. They found that gills are involved in a variety of ion transport processes, and the hepatopancreas is involved in immune responses. Kim et al. (2019) used the Illumina MiSeq platform to generate the transcriptome of the digestive gland in *Saxidomus purpuratus* and discovered that it performed functions related to innate immunity, digestion, and metabolism. Bertucci et al. (2018) performed RNA sequencing in *Corbicula fluminea,* a species in the Corbiculidae family that spread in freshwater and estuaries, exposed to water pollution. They found that genes for energy metabolism and survival in hypoxic conditions were differentially expressed depending on pollutants present in the environment *Corbicula fluminea* was living in.

The gills and hepatopancreas of *G. erosa* play an important role in environmental tolerance, resistance to pathogen infection, and metabolism. It is the focus of *G. erosa* research (Kim et al., 2019). As control group, elucidating the gene expression profiles and functions of muscle tissue is also important (Cappello et al., 2018).

High-quality transcripts are important for studying the biological functions of *G. erosa*. Due to the alternative gene splicing and a lack of genomic data, it is difficult to obtain accurate transcripts using second-generation sequencing methods. Third-generation sequencing (single molecule real-time sequencing, SMRT) has the advantage of longer sequencing reads, thus avoiding read errors caused by alternative splicing. PacBio’s long sequencing read length makes it ideal for identifying genetic subtypes, as well as novel genes and isoforms. However, PacBio has a high error rate.

In this study, full-length transcriptomes obtained by PacBio and Illumina sequencing were combined to construct the full-length transcriptome of *G. erosa*. Differential expression analysis was also performed between tissues taken from the gills, hepatopancreas, and muscle.

## Materials and Methods

### Collection and Preparation of Animal Material

In this study, animal samples for sequencing were obtained from mangrove wetlands in Beihai, Guangxi, China (21.57′N, 109.16′E), and animals for real-time quantitative PCR were obtained from estuary in Qinzhou, Guangxi, China (21.46′N, 108.37′E). No specific permissions were required for sample collection from these locations. Field studies did not involve endangered or protected species. Animal welfare and experimental procedures were carried out in accordance with the Guide for the Care and Use of Laboratory Animals and the ethical regulations of the Guangxi Mangrove Research Center. The shell length and weight of *G. erosa* samples were listed in Supplementary Table S1.

### RNA Extraction and Detection

Nine samples (three tissues-hepatopancreas, gill, and muscle, each with three biological replicates) were quickly separated on ice. Total RNA was extracted using Tiangen RNA preparation kits (Tiangen Biotech, Beijing, China) following the manufacturer’s protocol. The following methods were used to test the RNA quality. First, the extent of RNA degradation was estimated using agarose gel electrophoresis. Second, RNA purity (OD260/280), concentration, and absorption peaks were determined using a Nanodrop spectrophotometer. Third, RNA integrity was determined using Agilent 2,100 Bioanalyzer (Agilent Technologies, Santa Clara, CA, United States). Detection indexes included the RIN value, 28S/18S, baseline for spectra, and 5S peak. RNA samples were used to construct cDNA libraries. One microgram of each RNA sample from all samples was added together in a 1:1 ratio and used for PacBio single-molecule long-read sequencing. All nine samples were used for Illumina sequencing.

### Library Preparation and Sequencing

#### PacBio Library Preparation and Sequencing

A Clontech SMARTer^®^ PCR cDNA Synthesis Kit (Clontech Laboratories, 634,926, Mountain View, CA, United States) and the BluePippin Size Selection System protocol, as described by Pacific Biosciences (PN 100-092-800-03), were used to prepare the Iso-Seq library according to the Isoform Sequencing protocol (Iso-Seq).

#### Illumina Library Preparation and Sequencing

The Illumina library was prepared according to the Illumina (NEB, Ipswich, MA, United States) official NEBNext UltraTM RNA Library Prep Kit (E7530L) protocol. After the sample was qualified, the eukaryotic mRNA was enriched with magnetic beads with oligo (dT). Subsequently, fragmentation buffer was added to break the mRNA into short fragments. Using the mRNA as a template, single stranded cDNA was synthesized with six-base random primers (random hexamers). Buffer, dNTPs, DNA polymerase I, and RNase H were then added to synthesize double stranded cDNA. The double-stranded cDNA was purified with AMPure XP beads and poly(A) tails were added before sequencing adapters were selected by fragment size using AMPure XP beads. The final library was obtained by purification with AMPure XP beads and PCR amplification of the above sequence, after which the Illumina HiSeq 2000 platform was used to generate 150 bp paired-end reads.

### Bioinformatics Analysis

#### Error Correction Using Illumina Reads

Additional nucleotide errors in the consensus reads were corrected using Illumina RNA-seq data with LoRDEC software (Salmela and Rivals, 2014). The full-length transcriptome was calibrated using multiple methods to improve data accuracy. The methods used are as follows. 1) The same template was sequenced multiple times to perform in-hole correction for zero-mode waveguide holes to obtain a high-quality Circular Consensus (CCS). 2) Cluster multi-copy transcriptional sequencing data was used to ensure there was no redundancy, perform inter-hole correction of zero-mode waveguide holes, and obtain cluster consensus sequences. 3) Arrow software was used to calibrate the cluster consensus sequence using a non-full-length sequence, and thus establish a polished consensus sequence. 4) The polished consensus sequence derived from the second-generation data was refined using LoRDEC software (Salmela and Rivals, 2014), thus further improving sequencing accuracy. LoRDEC is a software which uses second generation data to correct the data generated by third generation PacBio sequencing. It employs mixed error correction, and is highly accurate, far more so than other correction software such as LSC (Au et al., 2012) and PacBioToCA (Koren et al., 2012). Any redundancy in the corrected consensus reads was removed using CD-HIT (Fu et al., 2012) (-c 0.95 -T 6 -G 0—aL 0.00 -aS 0.99) before obtaining the final transcripts for subsequent analysis. This software uses a heuristic algorithm to quickly find highly similar fragments among sequences.

#### Gene Functional Annotation

Gene functions were annotated using the following databases: NCBI non-redundant protein sequences (NR) (Li et al., 2002), protein family (Pfam) (Finn et al., 2016), Clusters of Orthologous Groups of proteins (KOG/COG) (Tatusov et al., 2000), Swiss-Prot (Bairoch and Apweiler, 2000), Kyoto Encyclopedia of Genes and Genomes Ortholog database (KEGG) (Kanehisa et al., 2004), and Gene Ontology (GO) (Ashburner et al., 2000). We used Diamond BLASTX software and an e-value of “1e-10” when performing NR, KOG, Swiss-Prot and KEGG database analysis. Hmmscan was used for family identification with Pfam database.

We used TransDecoder (v3.0.0) (Altschul et al., 1997) to predict coding sequence (CDs). The Pfam database was used to identify reliable potential CDs from the transcript sequence.

Long non-coding RNAs (lncRNAs) are a class of RNA molecules whose transcripts exceed 200 nt in length and do not code for proteins. We used the Coding–Non-Coding-Index (CNCI) (Sun et al., 2013), coding potential calculator (CPC) (Kang et al., 2017), Pfam-scan (Finn et al., 2016), and coding potential assessment tool (CPAT) (Wang et al., 2013) as tools to predict the coding potential of the transcripts. We used the CNCI with default parameters. Using the NCBI eukaryotes protein database, we set the e-value to “1e-10” in CPC. We translated each transcript in all three possible frames and used Pfam Scan to crossmatch with the Pfam database and identify sequences coding for any known protein family domains. All Pfam searches were performed using the default parameters of -E 0.001 --domE 0.001. The transcripts not predicted to have coding potential by all four tools above were excluded, and those without coding potential were candidate sets of lncRNAs. We further used the LncTar (Haas et al., 2013) (version 1.0, parameters: ndG value = − 0.1) as a tool to predict lncRNA-targeted transcripts.

#### Defining Alternative Splicing Events

We ran all-vs-all BLAST with high identity settings. BLAST alignments that met all the following criteria were classified as products of candidate alternative splicing (AS) events: 1) Two high-scoring segment pairs (HSPs) exist in the same alignment, with the same forward/reverse direction. 2) One sequence should be continuous or have only a small “overlap” size (smaller than 5 bp); the other one should be distinct, thus creating what is called an “AS Gap.” 3) The continuous sequence should almost completely align with the distinct sequence. 4) The AS gap should be longer than 100 bp and at least 100 bp away from the 3'/5′ end.

#### Gene Expression Quantification

Bowtie2 was used to compare the reads sequenced from each sample with the transcript sequences. Fragments per kilobase of transcript per million mapped reads (FPKM) is the number of reads per million reads that are aligned to a gene per kilobase length. It is a commonly used method for estimating gene expression levels in transcriptome sequencing data analysis. FPKM can eliminate the influence of differences in transcript length and sequencing amount on calculated expression. The calculated level of transcript expression can be directly used to compare transcript expression differences between samples. Gene expression levels were estimated by RNA-Seq by Expectation-Maximization (RSEM) (Li and Dewey, 2011) for each sample, as follows: 1) Clean data was mapped back onto the transcript sequences, 2) The read count for each transcript was obtained from the mapping results and 3) The read count was transformed to fragments per kilobase million (FPKM) (Zhao et al., 2021). Bowtie2 of the comparison software in RSEM was set to enter an end-to-end and sensitive mode, and the other parameters were set to default.

#### Gene Set Enrichment Analysis

We used Molecular Signatures DataBase (MSigDB) software (Liberzon et al., 2015) to measure the statistical enrichment of the genes. KEGG, GO, KOG, and Pfam databases were used to categorize transcripts into different gene classes. We then performed Fisher’s exact test on each class in the ordered genes or the user-specified gene list. *p* < 0.05 was the threshold which defined significant enrichment.

#### Gene Co-Expression Analysis

The weighted gene co-expression network analysis (WGCNA) (Langfelder and Horvath, 2008) approach is a popular method used to identify co-expressed and hub genes. WGCNA has been widely used to identify co-expressed gene modules and to find associations between modules and traits (Li et al., 2017). Based on the gene expression data, this software was used to analyze associations between modules and tissues (traits). In undirected networks, genes within the same module are highly related. After clustering genes into modules, each module was analyzed in two ways, which were described as follows: 1) Functional enrichment analysis was used to determine whether functional characteristics and traits that were identified were consistent with the aims of our research, and 2) Correlation analysis was conducted to determine the module that had the highest correlation with traits of interest. For all samples, we set power to 12, minModuleSize to 30, and maxBlockSize to the number of genes.

### Quantitative Real-Time PCR Analysis

Twelve genes were randomly chosen to validate the RNA-seq data. The animal samples used for Real-Time PCR analysis were collected from another location distinct from the samples using for sequencing. Four tissues-hepatopancreas, gill, muscle and the female gonad were quickly separated on ice. Total RNA for qRT-PCR were isolated from four tissues using the Tiangen RNA preparation kits (Tiangen Biotech, Beijing, China) as previously described. First-strand cDNA was reverse transcribed using the PrimeScriptTM RT reagent Kit with gDNA Eraser (Takara, Dalian, China). qRT-PCR was performed using TB Green Premix Ex Taq (Tli RNaseH Plus; Takara, Dalian, China). In the past experiment of our lab, to selected the suitable reference genes of different tissues in *G. erosa* by using qRT-PCR method, we compared four candidate genes, which were *β-actin, 18S rRNA, GAPDH* and *α-tubulin*, and the *α-tubulin* was the most stable expressed gene in different tissues. Thus, we use *α-tubulin* as the reference gene in this study. All primers of selected genes were designed using Primer3Plus and are listed in Supplementary Table S2. The conditions for qRT-PCR amplification were as follows: incubation at 95°C for 30 s, 40 cycles of 95°C for 5 s and 60°C for 34 s. The specificity of primer amplicons was tested using a melting curve analysis and PCR products were verified by sequencing. The amplification was carried out in a QuantStudio3 (ABI, United States) containing three biological replicates and three technical replicates.

### Transcriptome Coverage Assessment

The completeness of the transcriptome is difficult to assess due to the lack of a reference genome. The current common solution is to use the coverage of single-copy genes necessary for life to check the coverage of the transcriptome such as BUSCO (Benchmarking Universal Single-Copy Orthologs) (Manni et al., 2021). BUSCO (v5.3.0) is an open-source python software that evaluates genome assembly or transcripts based on gene evolution (with reference alignment). We use eukaryotes and mollusks as references, respectively, to evaluate our assembly results.

## Results

### Polymerase Read Statistics

The PacBio RS II used for SMRT sequencing contained 150,000 ZMWs (zero-mode waveguides) per cell. The reads were provided to the ZMW well for sequencing. One read (P1) per ZMW is valid. A total of 450,876 polymerase read sequences were obtained. Polymerase read fragments less than 50 bp and sequence accuracy less than 0.75 were excluded. The remaining reads were removed from the linker and the linker sequence was filtered out to obtain subreads (Table 1). Using this process, 3,847,562 subreads were obtained.

**TABLE 1:** Data statistics from Pacbio transcriptome sequencing.

cDNA Size	1–2K	2–3K	3–6K
Polymerase Reads	150,292	150,292	150,292
Polymerase Reads	107,731	115,408	108,719
Total Number of Subread Bases	3,099,865,656	3,208,845,527	3,050,228,963
Number of Subread	1,848,503	1,096,181	902,878
Subreads N50	1,696	3,050	3,835
Mean Subread length	1,676	2,927	3,378

### Transcript Classification

**FIGURE 1:**
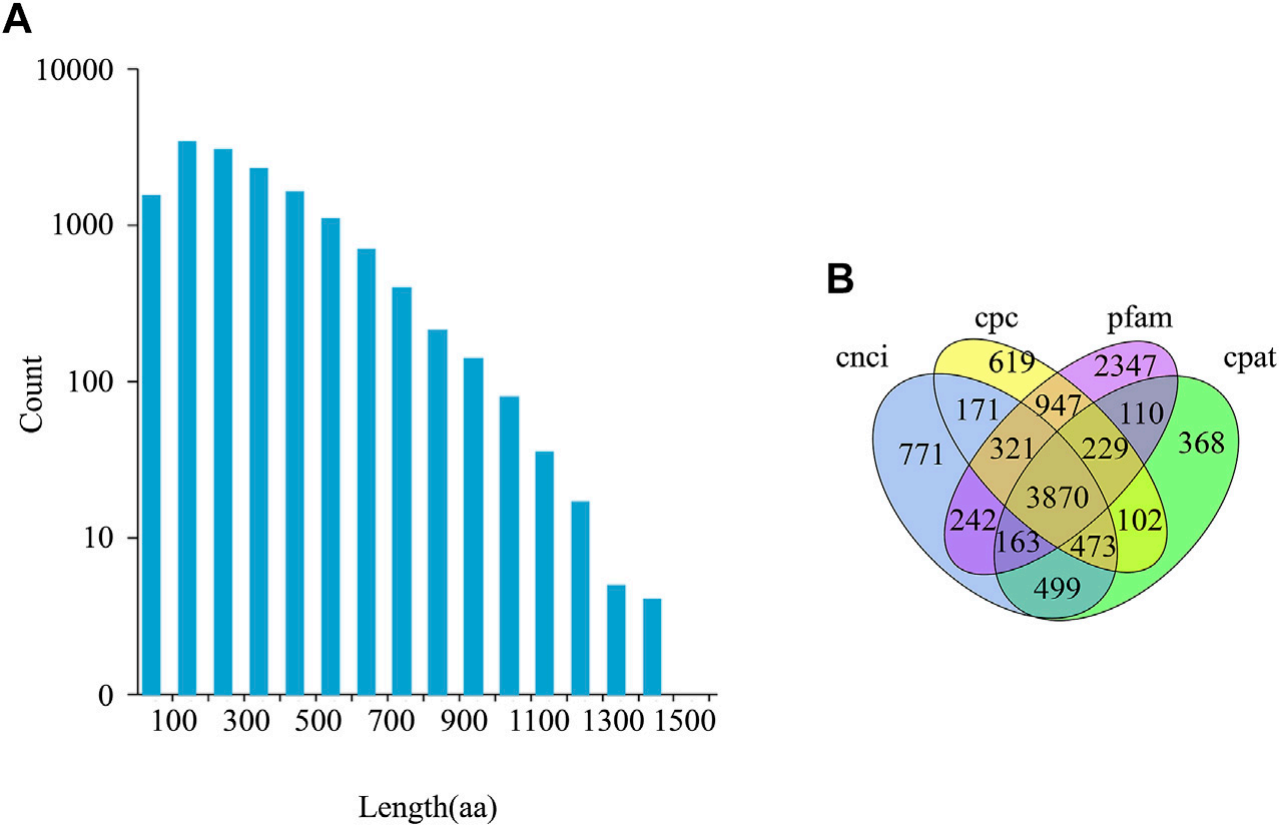
CDS (Coding Sequence) length distribution and lncRNA prediction. **(A)** The length distribution of the entire ORF region coded protein sequence predicted by CDS. aa stands for amino acid. **(B)** Venn diagram of lncRNA, predicted using CPC (Coding Potential Calculator), CNCI (Coding-Non-Coding Index), CPAT (Coding Potential Assessment Tool), and Pfam (Protein families).

Transcripts were classified as CDS or lncRNA sequences, based on their predicted protein coding ability. All transcript sequences are listed in Supplementary Table S5. A total of 19,411 ORFs were obtained, of which 14,496 were complete (Figure 1, Supplementary Table S3). A total of 3,870 lncRNA transcripts were identified, of which 978 contained target genes (Supplementary Table S3).

### Alternative Splicing

The pre-mRNA (pre-mRNA) generated by gene transcription has a variety of splice forms. Different exons are selected to produce different mature mRNAs, which are translated into different proteins, which constitute the diversity of biological traits. This post-transcriptional mRNA processing is called alternative splicing. A total of 31,881 consensus isoforms were obtained. 271 alternative splicing events were identified (Supplementary Table S3).

### Transcriptome Coverage

In eukaryotes we annotated 183 (71.8%) Complete BUSCOs (C), of which 118 (46.3%) Complete and single-copy BUSCOs (S) and 65 (25.5%) Complete and duplicated BUSCOs (D). In addition, there are 13 (5.1%) Fragmented BUSCOs (F) and 59 (23.1%) Missing BUSCOs (M).

In the Mollusca phylum we annotated 2,298 (43.4) Complete BUSCOs (C), of which, 1,526 (28.8%) Complete and single-copy BUSCOs (S) and 772 (14.6%) Complete and duplicated BUSCOs (D). In addition, there are 118 (2.2%) Fragmented BUSCOs (F) and 2,879 (54.4%) Missing BUSCOs (M).

Since this is transcriptome research and not all tissues, not all genes are expressed. The BUSCO completeness is relatively low compared to the genome (Schaarschmidt et al., 2020). The BUSCO completeness of our transcriptome is around 71%, which is similar to the European starling research (around 63%) (Stuart et al., 2021) by ISO-seq. The proportion of fragmented genes is very small (5.1%), indicating that our assembly results are relatively reliable.

### Gene Function Annotation

Due to the lack of corresponding genome annotation information for *G. erosa*, we annotated each transcript in seven databases. Genes were annotated by information of homologous proteins (NR, Swissprot), orthologous proteins (COG/KOG), protein domain information (Pfam), gene functions and involved pathways, biological processes, etc. (GO, KEGG). Eight databases were used to annotate gene functions: GO, KEGG, COG/KOG, Swissprot, NR, and Pfam. A total of 17,541 genes were annotated (Table 2; Supplementary Table S3). Among them, the NR database annotated the most transcripts (16,985), while the transcripts annotated by GO were relatively few (6,002).

**TABLE 2:** Annotated transcript number statistics table.

Database	Number of Annotated Transcripts
All	17,541
GO	6,002
KEGG	8,329
KOG	11,384
Pfam	14,207
Swissprot	9,666
COG	5,421
NR	16,985

“Database” indicates the database used for functional annotations. “Annotated number” refers to the number of transcripts used to obtain this annotation from the corresponding database.

### Gene Expression Analysis

**FIGURE 2:**
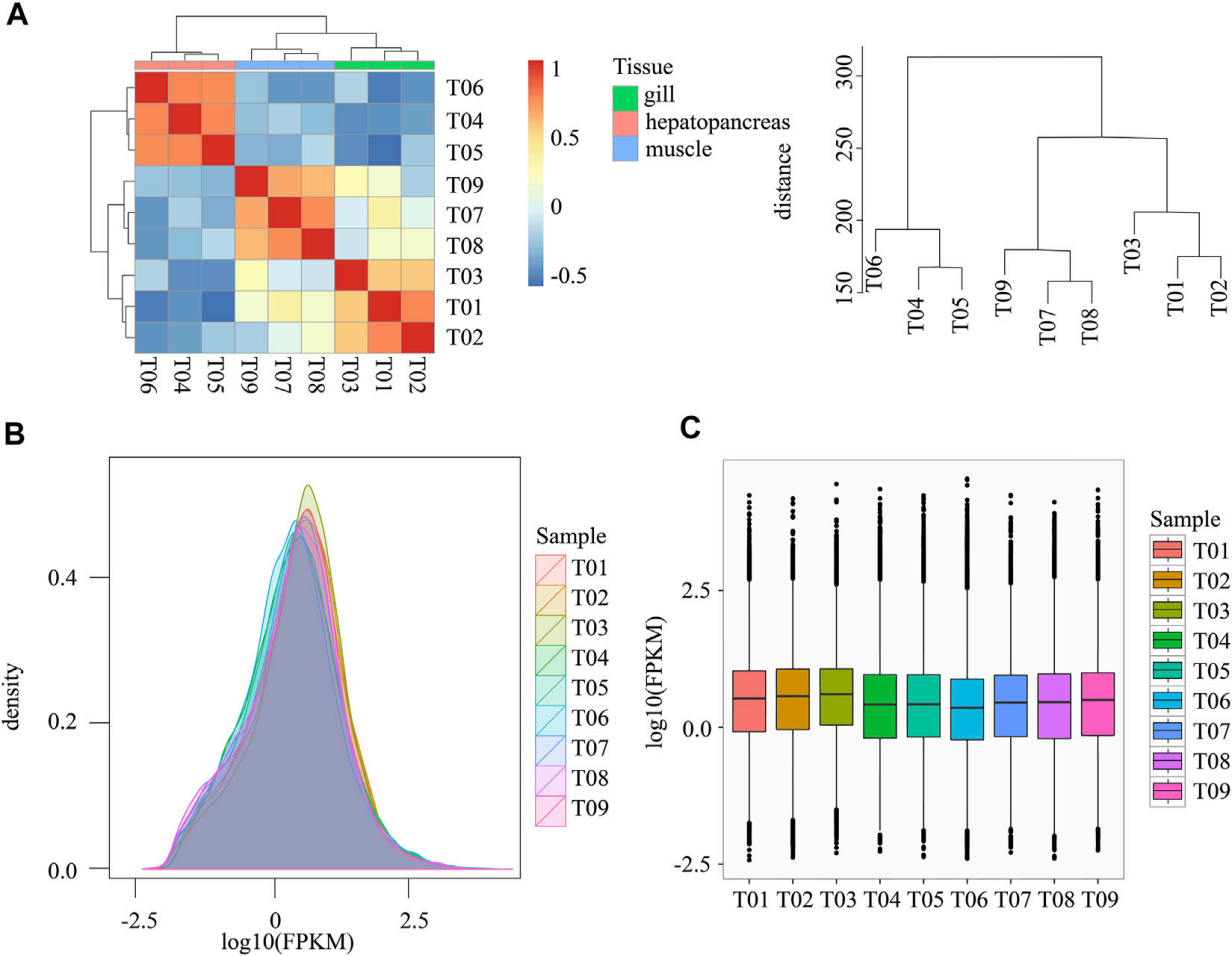
Statistics regarding gene expression. **(A)** Hierarchical clustering and heat map of all samples. The right plot shows the clustering distance between samples. **(B)** Density distribution of expression values from all samples. **(C)** Box plot expression values from all samples. Note: FPKM: Fragments Per Kilobase of transcript per Million mapped reads. Samples T01-T03 belong to gill, T04-T06 belong to hepatopancreas, T07-T09 belong to muscle.

Bowtie2 was used to compare reads obtained by sequencing each sample with the transcript sequence (Table 3). Final quantitation of transcript expression was obtained and converted to FPKM (Figure 2; Supplementary Table S4). The expression levels of the randomly selected genes using qRT-PCR analysis matched these of high throughput sequencing data.

**TABLE 3:** Comparison of results derived from second-generation sequencing vs. full-length transcripts.

BMK-ID	Total reads	Mapped Reads (%)	Unique Mapped Reads (%)	Multi Mapped Reads (%)
T01	31,520,554	18,157,222 (57.60%)	5,793,284 (31.91%)	12,363,938 (68.09%)
T02	32,752,380	17,857,615 (54.52%)	5,660,251 (31.70%)	12,197,364 (68.30%)
T03	27,667,629	16,465,587 (59.51%)	5,251,710 (31.90%)	11,213,877 (68.10%)
T04	32,933,308	18,811,133 (57.12%)	6,634,051 (35.27%)	12,177,082 (64.73%)
T05	30,115,694	17,211,031 (57.15%)	6,131,141 (35.62%)	11,079,890 (64.38%)
T06	29,407,809	18,630,763 (63.35%)	6,329,039 (33.97%)	12,301,724 (66.03%)
T07	28,991,037	17,665,871 (60.94%)	4,681,671 (26.50%)	12,984,200 (73.50%)
T08	31,697,911	18,775,882 (59.23%)	5,462,242 (29.09%)	13,313,640 (70.91%)
T09	30,691,848	18,716,281 (60.98%)	5,519,848 (29.49%)	13,196,433 (70.51%)

BMK-ID, refers to sample id. Total reads refer to total reads for each sample. Mapped reads refer to the percentage of reads that aligned to the reference transcript. Unique mapped reads refer to the percentage of mapped reads that were unique. Multi-mapped reads refer to the percentage of mapped reads that were not unique.

We adopted the Pearson Correlation Coefficient r as the correlation evaluation index of biological replicate correlation. The value range of r is between −1 and 1, greater than 0 indicates a positive correlation, and less than 0 indicates a negative correlation. Larger absolute values indicate stronger correlations. The gene expression abundances of the nine samples had similar distributions (Figure 2B). Different tissues expressed significantly different genes (Figures 2A,C). Figure 2A shows that differences between tissues are significantly greater than differences within tissues. The difference in expression of hepatopancreas was greater than that of other tissues.

#### Differential Expression Statistics

From the gene expression correlation analysis (Figure 2A), we found significant differences between different tissues. In the process of differential expression genes (DEGs) analysis, we can do differential analysis between any two tissues. In this paper, we focus on tissue-specific expression. We take the gill, hepatopancreas, and muscle as case groups, respectively, other tissues were used as the control group for DEGs analysis between tissues. We adopted Deseq2 as a differential expression analysis tool.

**FIGURE 3:**
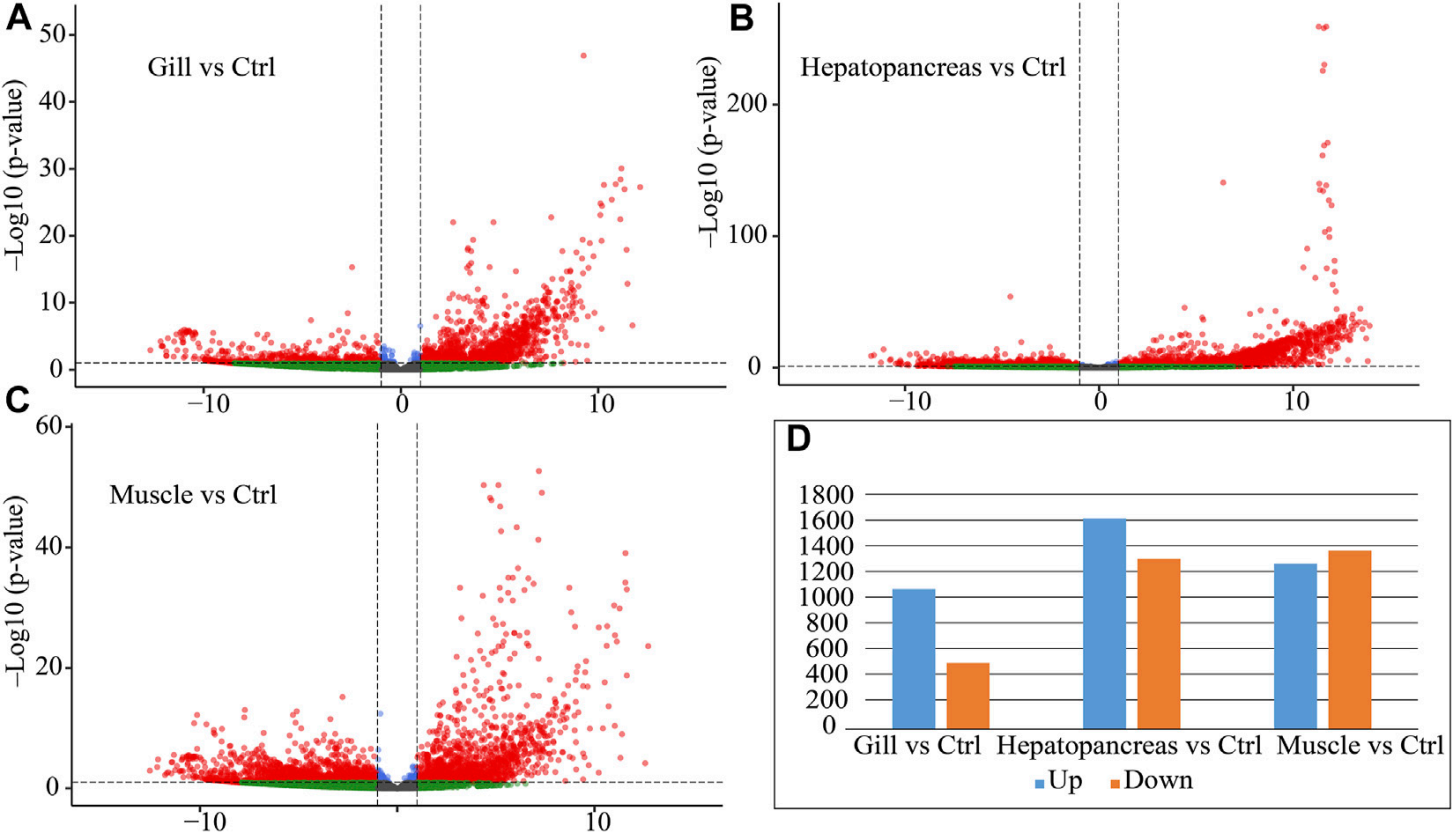
Differential expression statistics for muscle, hepatopancreas and gill tissue. **(A)** Volcano plot of differential expression between gill and control (hepatopancreas and muscle). **(B)** Volcano plot of differential expression between hepatopancreas and control (gill and muscle). **(C)** Volcano plot of differential expression between muscle and control (gill and hepatopancreas). **(D)** Differential expression statistics for each tissue. Note: Ctrl: control; Up: upregulated genes; Down: downregulated genes.

A total of 22,749 transcripts were quantitatively expressed (Supplementary Table S5). Among them, 2,981 genes were differentially expressed between the hepatopancreas and other tissues (gill and muscle), 1,614 genes were differentially expressed between gills and other tissues (hepatopancreas and muscle), and 2,692 genes were differentially expressed between muscle and other tissues (hepatopancreas and gill) (Figure 3; Supplementary Table S5).

We found that DEGs have the following characteristics. 1. Hepatopancreas had more DEGs, and their fold changes were also higher (Figure 3B, UP: 1,649, DOWN: 1,332). 2. Most of the DEGs in gills were up-regulated (UP: 1,096, DOWN: 518). 3. The number of up-regulated and down-regulated genes in muscle are roughly the same (UP: 1,295, DOWN: 1,397). These results suggest that hepatopancreas and gills may perform specific functions.

#### Enrichment Analysis and Analysis of Gene Co-Expression Between Tissues

**FIGURE 4:**
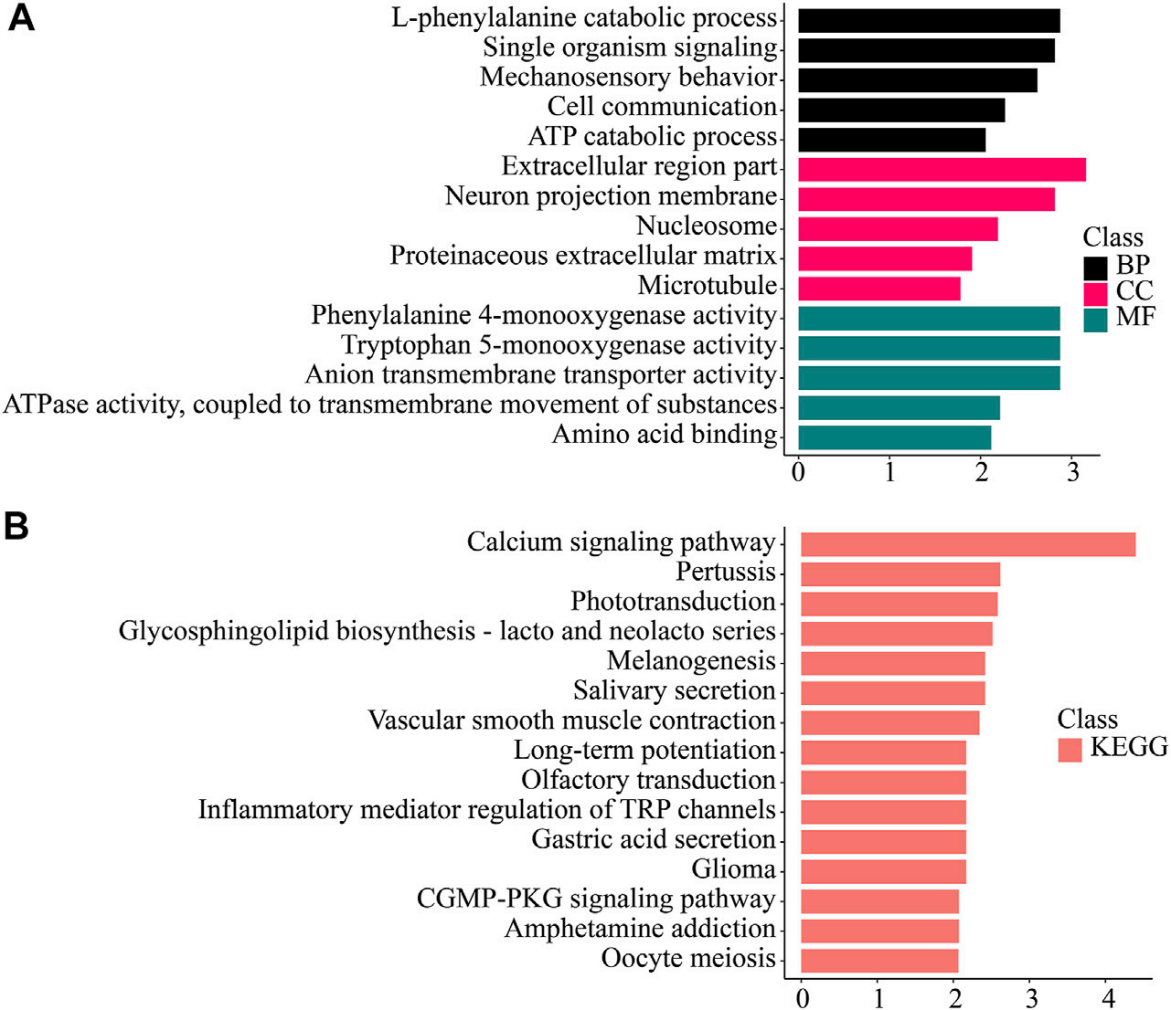
Enrichment analysis of gill-specific differentially expressed genes. **(A)** GO enrichment. BP: Biological process. CC: Cellular component. MF: Molecular function. **(B)** KEGG enrichment. KEGG: Kyoto Encyclopedia of Genes and Genomes; GO: Gene Ontology.

**FIGURE 5:**
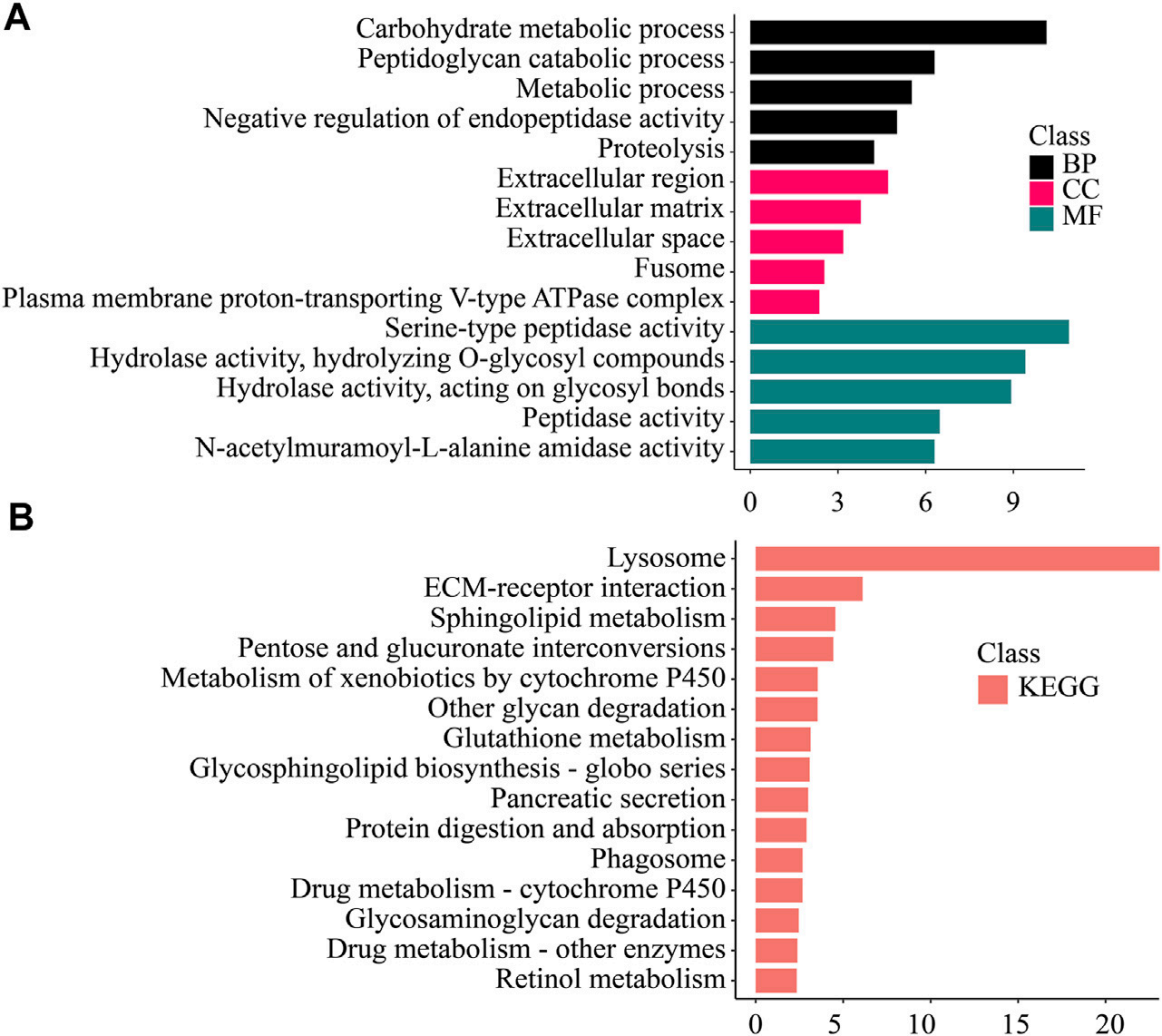
Enrichment analysis of Hepatopancreas -specific differentially expressed genes. **(A)** GO enrichment. BP: Biological process. CC: Cellular component. MF: Molecular function. **(B)** KEGG enrichment. KEGG: Kyoto Encyclopedia of Genes and Genomes; GO: Gene Ontology.

**FIGURE 6:**
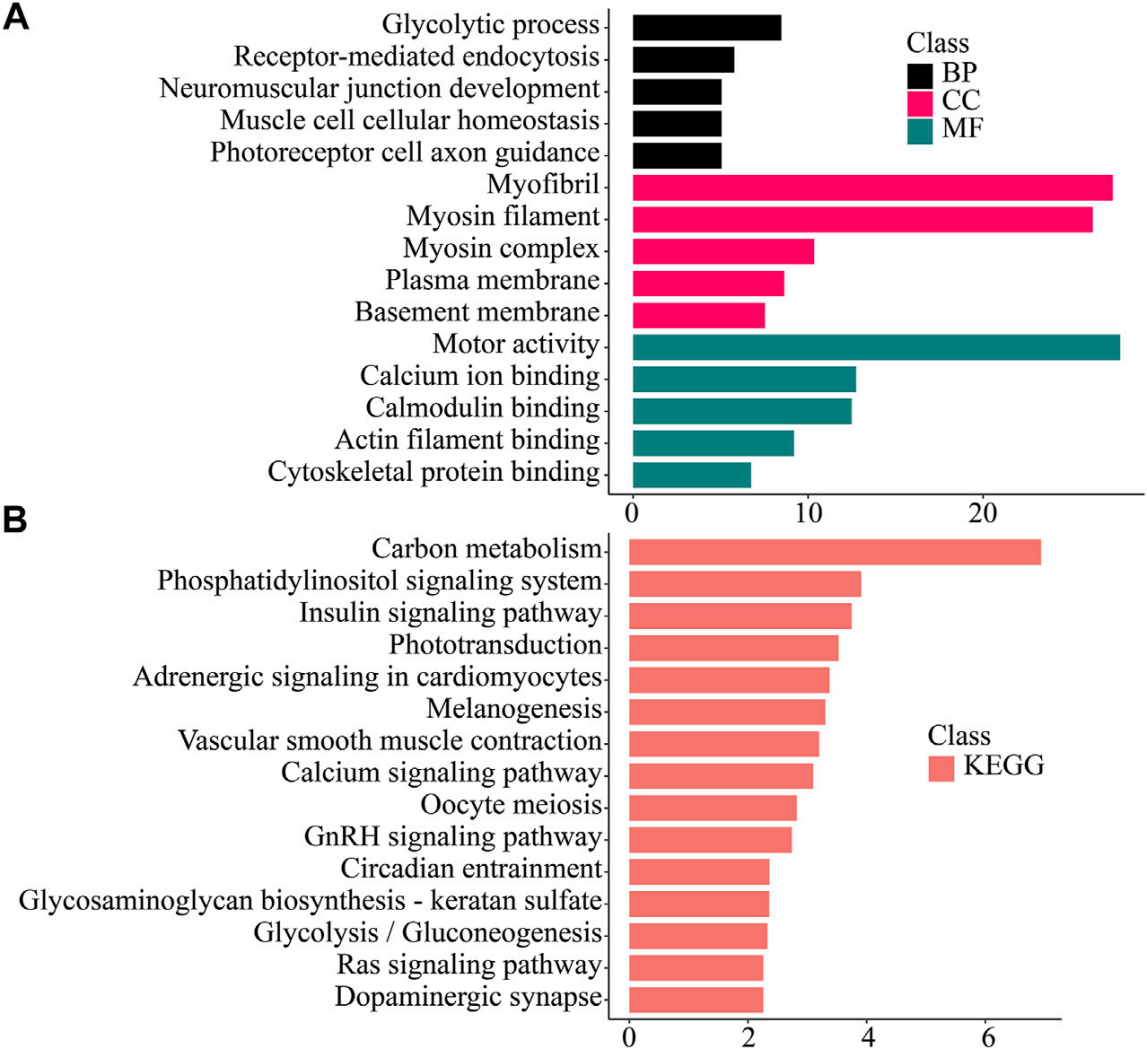
Enrichment analysis of Muscle -specific differentially expressed genes. **(A)** GO enrichment. BP: Biological process. CC: Cellular component. MF: Molecular function. **(B)** KEGG enrichment. KEGG: Kyoto Encyclopedia of Genes and Genomes; GO: Gene Ontology.

To study the function of the differentially expressed genes (DEGs), we performed GO, KEGG, KOG/COG, and Pfam enrichment analysis on the differentially expressed genes in each tissue (Figures 4–6; Supplementary Table S5).

We found that genes significantly up-regulated in gills were enriched in Mechanosensory behavior, l-phenylalanine catabolic process, Phenylalanine 4-monooxygenase activity and other processes and functions. In the KEGG database, Calcium signaling pathway, Phototransduction, Glycosphingolipid biosynthesis-lacto and neolacto series pathway and other pathways were enriched.

Genes that were significantly up-regulated in the hepatopancreas were enriched in various metabolic processes (Carbohydrate metabolic process, Peptidoglycan catabolic process, Proteolysis, Hydrolase activity, Hydrolyzing O-glycosyl compounds). Variety of digestive and metabolic activities (Sphingolipid metabolism, Pancreatic secretion, Protein digestion and absorption), detoxification (Drug metabolism—cytochrome P450, Drug metabolism—other enzymes etc.), xenobiotic degradation (Lysosome, Metabolism of xenobiotics by cytochrome P450), and other pathways were significantly enriched.

In muscle tissue, significantly up-regulated genes were enriched in functions related to muscle such as muscle cell cellular homeostasis, motor activity, calcium ion binding, actin filament binding. In the KEGG pathway, it was related to carbohydrate metabolism (Carbon metabolism, Glycolysis/Gluconeogenesis), muscle activity (Vascular smooth muscle contraction), nerve conduction (Adrenergic signaling in cardiomyocytes, Dopaminergic synapse), and intracellular signal transduction (Calcium signaling pathway, Phosphatidylinositol signaling system, Ras signaling pathway) related pathways were significantly enriched.

The detailed functions involved in the gills, hepatopancreas, muscle, were shown in the Discussion section.

**FIGURE 7:**
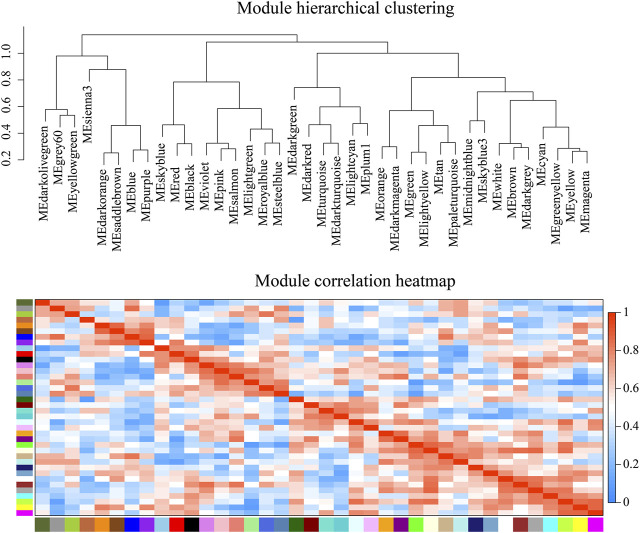
Weighted gene co-expression network analysis (WGCNA) module hierarchical clustering. The value range of correlation is 0–1.

**FIGURE 8:**
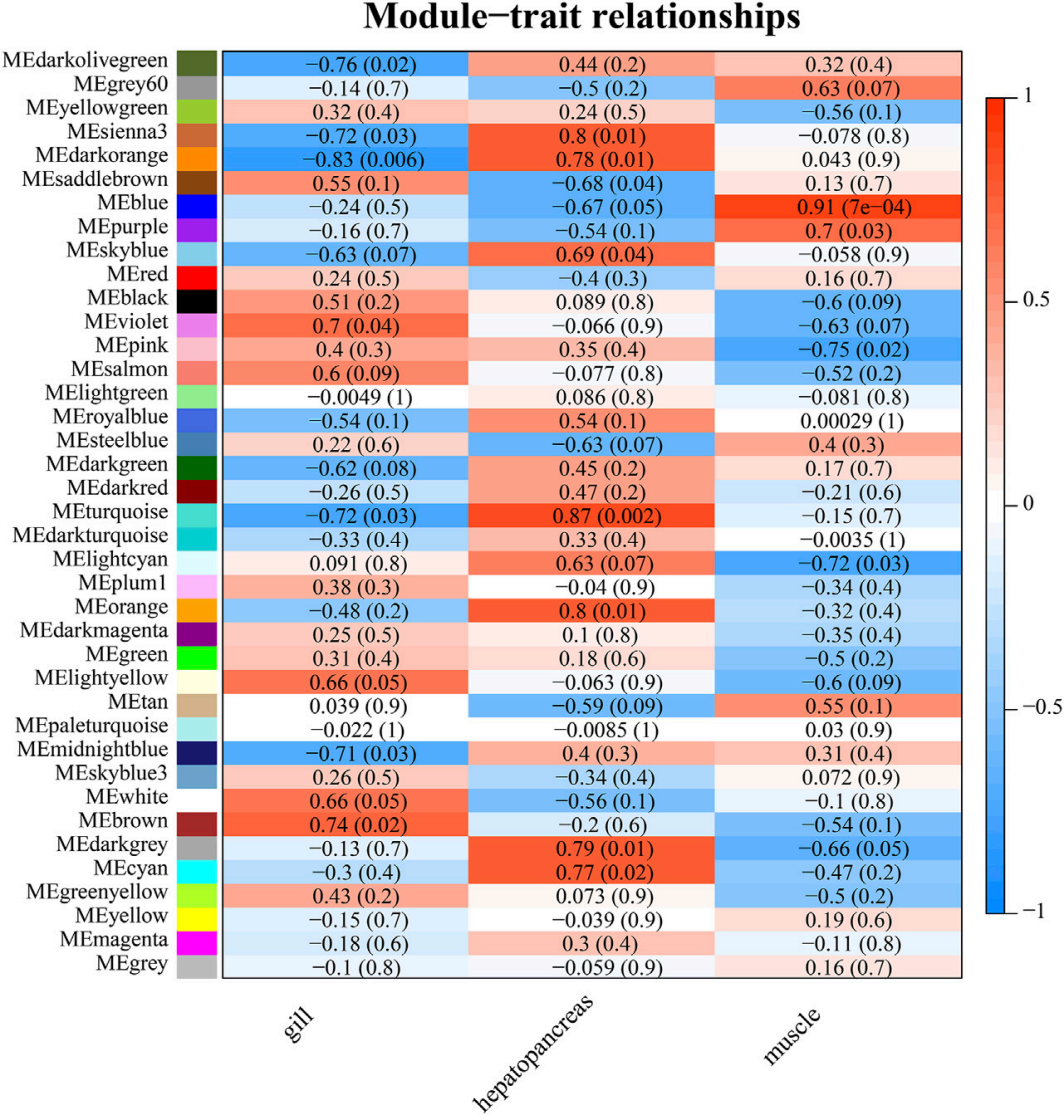
Heat map of module-trait relationships. The *p*-value is indicated in parentheses. The correlation method is the person correlation coefficient. The value range is −1 to 1. The larger the absolute value, the stronger the correlation. A value of less than 0 indicates a negative correlation.

WGCNA analysis can discover gene modules with similar expression patterns, as well as correlations between gene modules and phenotypic properties. By this approach, we identified 38 modules. There were 3,897 genes in the largest module, and 37 genes in the smallest module. The average amount of genes within a module was 588. (Figure 7; Supplementary Table S6). Module-tissue correlation analysis revealed that two modules were significantly positively correlated with gill tissue (*p* < 0.05); eight with hepatopancreas tissue, and two with muscle tissue (Figure 8; Supplementary Table S6).

### Validation of Differentially Expressed Transcripts of Tissues

**FIGURE 9:**
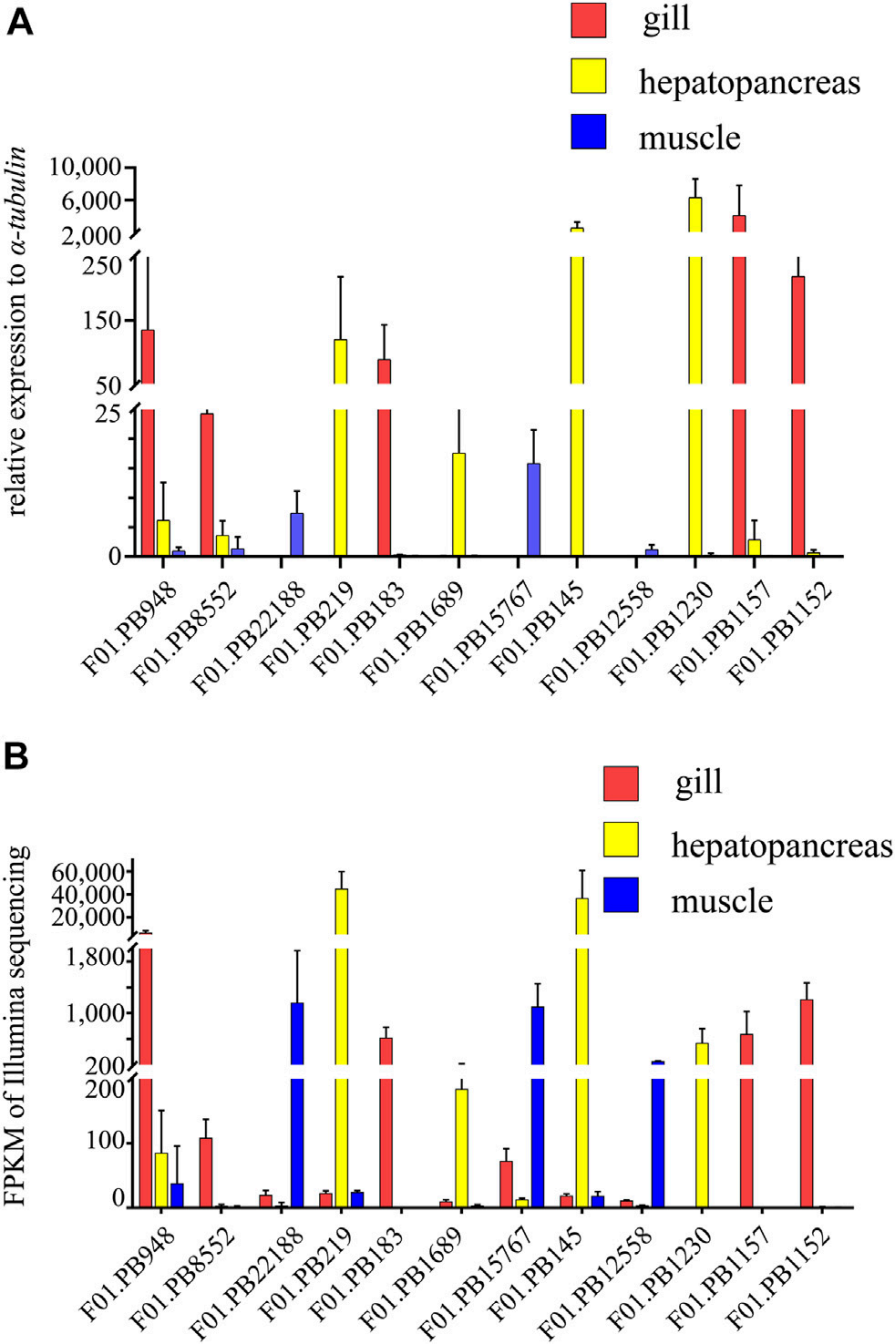
The relative expression levels and FPKM of selected twelve genes. **(A)** Relative expression levels of selected twelve genes in three tissues of *G. erosa* by qRT-PCR. 2^−ΔΔCt^ values (normalized to housekeeping gene *α-tubulin*) represented the expression of genes in three tissues (gill, hepatopancreas and muscle) of *G. erosa*. **(B)** Graphical representation of FPKM value of the twelve genes from Illumina sequencing results. Bars represent the average results of triplicate reactions for each tissue. Error bars represent standard error of the mean (SEM) among three biological replicates.

Twelve genes among annotated or un-annotated DEGs were randomly selected, and real-time PCR (qRT-PCR) analysis was conducted to validate the differentially expressed transcripts. The total RNA extracted from four tissues of *G. erosa* samples was used for Real-Time PCR analysis. Data were quantified using the 2^−ΔΔCt^ method based on Ct Values. All the samples were normalized to the housekeeping gene *α-tubulin*, and the concerned three tissues were normalized to the female gonad. The relative expression levels of twelve genes in the three tissues and the FPKM of these genes from RNA-seq results were showed in (Figure 9). All the twelve genes showed consistent qRT-PCR expression patterns as the high throughput sequencing data. The results showed that the results of RNA-seq technique were credible.

## Discussion

*Geloina erosa* plays an important role in mangrove ecology, however, compared to well-studied economic mussels, little attention has been paid to it. Full-length cDNA sequences are basic resources for molecular studies. In this study, full-length transcriptomes of *G. erosa* was sequenced by SMRT and Illumina sequencing were applied for studying the tissue-specific physiological functions. Additionally, the expression levels of randomly selected genes using qRT-PCR analysis validated the credibility of high throughput sequencing data.

### Gill-Specific Genes and Functions

The gills evolve from the endoderm (Green et al., 2015; Janvier, 2015; He et al., 2020). The gill is the first barrier in the body of bivalves and is the main point of interaction between the organism and the environment (Cannuel et al., 2009). It contains many filaments that increase its surface area to allow constant exchange of substances between the environment and the body. Because of this, it is exposed to many environmental stresses such as microorganisms, pollutants, pH and changes in salinity (Moreira et al., 2015). In most bivalves, gills are also involved in respiration and cilia suspension feeding. Mussels use the cilia on their gills to pump water through the inhalant siphon into the mantle cavity, where ciliary action along the gills draws water over these filter-feeding organs (Galbraith et al., 2009). Biological processes enriched in the gill may be related to this filtering function, including mechanosensory behavior (Bezares-Calderón et al., 2020) and cell communication. Cellular components necessary for filtering include a proteinaceous extracellular matrix, microtubules, and kinesin complexes. Molecular functions necessary for filtering include structural components of the cytoskeleton and microtubule motor activity (Gäde, 1981; Dobrzhanskaya et al., 2010).

We found that biological processes related to metabolism are enriched in the gills of *G. erosa*, such as inositol biosynthetic and phospholipid biosynthetic processes. In gene co-expression (WGCNA) analysis, we found that the MEbrown module was positively correlated with the gills. After enrichment analysis of all genes in this module, the GO items that were significantly enriched included glycosaminoglycan biosynthesis-keratan sulfate, glycosphingolipid biosynthesis-lacto, and neolacto series.

Gills may play an important role in identifying pathogens and activating immunity (Zhao et al., 2017; Saco et al., 2020). Immune activation leads to activation of phagocytes, generation of reactive oxygen species (ROS), and secretion of immune effectors and cytokines (Soudant et al., 2013). Previous research has shown that gills express many transcripts related to the recognition of antigens (Wang et al., 2008; Gerdol et al., 2011; Romero et al., 2011; Moreira et al., 2015). Monooxygenase is involved in immune responses in *Caenorhabditis elegans* (Wani et al., 2021). In the transcriptome of gill, we found active monooxygenase (phenylalanine 4-monooxygenase activity, tryptophan 5-monooxygenase activity) enriched in GO Molecular function (MF) gene set. We also found that cytochrome P450 CYP2 subfamily were enriched in the KOG database, and cytochrome P450 and multicopper oxidase were enriched in the Pfam database. The glutaredoxin protein is involved in the detoxification of ROS, and in this study, gene F01. PB8852 is homologous to the *glutaredoxin* of a kind of abalone, *Haliotis diversicolor supertexta* (Nam et al., 2016). The result of qRT-PCR showed that, expression of gene F01. PB8852 was abundant in gills. Taken together, these results indicate that gills play an important role in immunity and are likely the first line of defense against infection in *G. erosa*.

Furthermore, we found genes involved in multiple pathways and functions related to neural activity. Using the KEGG database, we identified genes involved in the neurotrophin signaling pathway, long-term potentiation, and dopaminergic. Using the Pfam database, we identified genes related to neurotransmitter gated transmembrane ion channels. Compared with the hepatopancreas and muscle, gill metabolite analysis revealed the presence of more neurotransmitters, such as acetylcholine (Cappello et al., 2018). By subcellular localization, we discovered genes related to neuron projection membranes, glial cell migration, and other neural pathways. In the Pfam database, the region corresponding to transmembrane neurotransmitter-gated ion channels was significantly enriched. The acetylcholine receptor region was enriched in the KOG database (Supplementary Table S3) (Jørgensen, 1974; Cappello et al., 2015). Together, these results suggest that gill activity is under the control of the nervous system (Carroll and Catapane, 2007).

### Hepatopancreatic Specific Genes and Functions

The hepatopancreas is an organ with combined functions of the liver and pancreas (Xu et al., 2021). The vertebrate hepatopancreas is also called the mid-intestinal gland (Rőszer, 2014). Research suggests that the invertebrate hepatopancreas is involved in biological processes such as glycogen, calcium, and lipid storage, as well as excretion of nitrogen metabolites. (Sumner, 1965; Rőszer, 2014). However, the invertebrate hepatopancreas is not homologous to the vertebrate hepatopancreas, but rather to the vertebrate bilaterian midguts (Hashimshony et al., 2015; Steinmetz et al., 2017). The digestive gland-like system of invertebrates is controlled by the Pdx gene, which belongs to the ParaHox cluster (Fritsch et al., 2016; He et al., 2020).

A variety of metabolic processes, such as metabolism of carbohydrates and lipids, and peptidoglycan catabolism, were enriched in this study. In molecular function (MF), a variety of hydrolase activities were enriched, such as hydrolyzation of O-glycosyl compounds and glycosyl bonds. Oxidoreductase activity and peptidase activity was also enriched. In the KEGG database, we identified genes related to pancreatic secretion, pathways involving the lysosome and phagosome, drug metabolism, cytochrome P450, lipid metabolism (sphingolipid metabolism, glycosphingolipid biosynthesis-globo series), amino acid metabolism (glycine, serine, and threonine metabolism), sugar metabolism (pentose and glucuronate interconversions, other glycan degradation, starch and sucrose metabolism), and vitamin ligand metabolism (folate biosynthesis, arachidonic acid metabolism, pantothenate and CoA biosynthesis). A variety of enzymes related to protein metabolism were found in the KOG database: trypsin, zinc carboxypeptidase, cysteine proteinase cathepsin L, hydrolytic enzymes of the alpha/beta hydrolase fold, glutamate/aspartate, and neutral acid transporters. In the Pfam database, we found that the papain family cysteine protease, trypsin, glycosyl hydrolase family 9, peptidase C1-like family, and peptidase family M13 were related to protein metabolism. Lipase is involved in lipid metabolism. After WGCNA modular analysis, the MEturquoise module was found to be positively correlated with the hepatopancreas. This module was enriched in pentose and glucuronate interconversions, glycine, serine, and threonine metabolism, pancreatic secretion, protein digestion, and absorption. GO analysis also revealed pathways related to carbohydrate, protein, and lipid metabolism. We also found functions and pathways related to chitin metabolism, such as the chitin-binding peritrophin-A domain, chitinase.

The hepatopancreas is an important organ involved in the immune response, as well as responses to oxidative and heat stress in mollusks and crustaceans (Rőszer, 2014). Among the differentially expressed genes in the hepatopancreas, many belong to the monooxygenase family (Moore et al., 1980), such as peptidylglycine alpha-amidating monooxygenase, beta, beta-carotene 15,15′-dioxygenase and related enzymes, and copper type II ascorbate-dependent monooxygenase. The result of qRT-PCR showed that, expression of gene F01. PB219 and F01. PB145 were abundant in hepatopancreas. Gene F01. PB219 is homologous to *lysozyme* of a clam, *Mactra quadrangularis*, and the lysozyme protein has antibacterial activity against a number of bacterial species. Gene F01. PB145 is homologous to the *defensin* of a kind of mussel, *Dreissena polymorpha*, and the defensins are members of a large family of cationic antimicrobial peptides that form an essential element of innate immunity (Gerdol et al., 2020). Taken together, these results suggest that the hepatopancreas plays an important role in metabolism, immune response, and detoxification (Rőszer, 2014).

### Muscle-Specific Genes and Functions

Bivalve is an important fishery commodity, and muscle is the main edible part of bivalve organisms. Bivalve muscle has a high collagen and low fat content, and is rich in mineral elements (Tabakaeva et al., 2018).

Previous analysis of metabolite content in *G. erosa* has revealed that muscle tissue is far richer in energy metabolites than hepatopancreas or gill tissue. (Cappello et al., 2018). We found that biological processes related to energy metabolism include GTP catabolism, oxidation-reduction reactions, glycolytic processes, and the tricarboxylic acid cycle. Cellular components related to energy metabolism, include mitochondrial respiratory chain complex II. Molecular functions related to energy metabolism include cytochrome-b5 reductase activity, NAD(P)H involvement and malate dehydrogenase activity. We found KEGG pathway related energy metabolism including carbon metabolism, insulin signaling pathway, and glycolysis/gluconeogenesis. In WGCNA analysis, the MEblue module, which is enriched in functions related to glucose metabolism and glycolytic processes, was positively correlated with muscle tissue.

Previous studies have shown that muscles express many myofibril and calcium-related proteins (Moreira et al., 2015). In the current study, many functions related to muscle movement and control have been found to be enriched in *G. erosa*, such as neuromuscular junction development, cellular calcium ion homeostasis, myofibril assembly, striated muscle myosin thick filament assembly, actin nucleation, actin filament severing and capping, and synaptic transmission. Cellular components related to muscle function include myofibrils, myosin filaments, myosin complexes, postsynaptic membranes, axons, Z discs, and myosin IV and V complexes. Molecular functions related to muscle function include motor activity, calcium ion binding, calmodulin binding, actin binding, and neurotransmitter sodium symporter activity. In WGCNA analysis, the MEblue module, which was positively correlated with muscle, was also enriched in adrenergic signaling in cardiomyocytes, vascular smooth muscle contraction, actin crosslink formation, and synaptic growth at the neuromuscular junction. The result of qRT-PCR verified the reliability of sequencing result. The genes F01.PB22188, F01.PB15767 and F01.PB12558 with highly relative expression were respectively homologous to *paramyosin-3*, *myosin heavy chain* and *Filamin-A*.

We also identified pathways and functions related to photosensitivity. Biological processes related to photosensitivity include photoreceptor cell axon guidance, metarhodopsin inactivation, regulation of light-activated channel activity, and bioluminescence. The MEblue module was found to be enriched for functions related to photosensitivity, including phototransduction, photoreceptor activity, bioluminescence, and photoreceptor cell axon guidance.

The findings can be used to study the tissue-specific functions of *G. erosa* under natural conditions. The full-length transcript data provided by this study will provide a reliable and highly valuable resource for further *G. erosa* alternative splicing and protein structure research. Furthermore, this study may in the future act as a reference point for *G. erosa* transcriptomics. It could be used as a control for future studies which investigate gene expression in *G. erosa* under abnormal environmental stresses and other conditions. However, there are several disadvantages in our study. Firstly, our results are for gene expression in different tissues under normal conditions, which may be different for transcripts under other conditions such as environmental stress. Secondly, our results are based on bulk RNA-seq results with limited resolution. In the future, we will use single cell sequencing to quantify the gene expression of *G. erosa*.

## Data Availability

The datasets generated for this study can be found in the National Center for Biotechnology Information (NCBI) in Bioproject: PRJNA544778, with ID: SAMN21552671 to SAMN21552679 (T01 to T09). All annotation data (Supplementary Table S3), full-length sequence of transcripts (Supplementary Data Sheet 1.ZIP), expression data (Supplementary Table S4), and enrichment analysis results (Supplementary Table S5, Supplementary Table S6) are shown in the Supplementary Materials.
